# Impact of Bacterial Fermentation on Phytochemical Profile and Bioactivity of *Calendula officinalis* and *Echinacea purpurea* Extracts

**DOI:** 10.1155/ijfo/3514246

**Published:** 2026-05-08

**Authors:** Sofia Massaro, Jacopo Sica, Gloria Ghion, Simone Vincenzi, Davide Porcellato, Viviana Corich, Chiara Nadai, Alessio Giacomini

**Affiliations:** ^1^ Department of Agronomy Food Natural Resources Animals and Environment (DAFNAE), University of Padova, Legnaro, Province of Padua, Italy, unipd.it; ^2^ Interdepartmental Centre for Research in Viticulture and Enology (CIRVE), University of Padova, Conegliano, Treviso, Italy, unipd.it; ^3^ Faculty of Chemistry, Biotechnology and Food Science, Norwegian University of Life Sciences, Ås, Norway, nmbu.no; ^4^ Department of Land, Environment, Agriculture and Forestry (TESAF), University of Padova, Legnaro, Province of Padua, Italy, unipd.it

**Keywords:** calendula, echinacea, fermentation, functional properties, lactic acid bacteria

## Abstract

The increasing consumer attention toward plant‐based products having health‐promoting benefits has drawn attention to medicinal herbs such as *Calendula officinalis* and *Echinacea purpurea*, that are abundant in bioactive compounds exhibiting anti‐inflammatory, antimicrobial and antioxidant properties. In this work, four bacterial strains, *Lactiplantibacillus plantarum* 299 V, *Pediococcus acidilactici* IRZ12B, *Lacticaseibacillus rhamnosus* GG, and *Bacillus subtilis* natto, were used to ferment aqueous extracts of *C. officinalis* and *E. purpurea*. All tested strains exhibited robust growth in the herbal substrates, resulting in a marked drop in pH and increased microbial counts. Fermentation enhanced sugar utilization, stimulated organic acid synthesis, and reduced antinutritional compounds such as tannins, while improving the availability of certain minerals. After fermentation, extracts showed an increased total phenolic level, stronger antioxidant activity, and increased antimicrobial effects relative to unfermented controls. These findings indicate that bacterial fermentation, especially performed by lactic acid bacteria, can substantially augment the functional and phytochemical characteristics of *C*. *officinalis* and *E. purpurea* extracts. This approach supports the development of innovative plant‐based fermented nutraceuticals with therapeutic potential. Moreover, the optimized fermentation process offers a strategy for incorporating fermented *C*. *officinalis* and *E*. *purpurea* extracts into plant‐based food products, satisfying the increasing interest of consumers for nutrient‐rich, health‐oriented foods.

## 1. Introduction

In recent years, consumer interest has increasingly shifted toward plant‐based diets, motivated not only by their recognized health advantages but also by concerns over environmental sustainability and ethical aspects concerning animal welfare [1]. Many modern consumers are particularly attracted to the potential of plant‐based diets to support longevity and enhance overall well‐being [2]. This trend highlights a wider shift toward functional foods that go beyond basic nutrition, offering benefits that may help prevent disease and support overall health [1]. Plant‐derived foods are naturally abundant in bioactive compounds, which exhibit diverse biological effects and may help to decrease the risk of chronic problems such as certain cancers, cardiovascular disease, and diabetes [3].

The plant kingdom provides an extensive array of species containing bioactive molecules with substantial medicinal potential. These compounds have been isolated, characterized, and applied in a variety of contexts, particularly in pharmaceutical development [4, 5]. In the food sector, medicinal plant extracts have gained attention due to their long‐recognized health‐promoting properties, including traditional applications in managing infections and disorders affecting the skin, respiratory tract, and digestive system [4]. Among these, species from the genera *Calendula* and *Echinacea* are notable for their wound‐healing capabilities, as well as a range of other bioactivities, such as antioxidant, anti‐inflammatory, antimicrobial, and anticancer effects.

*Calendula officinalis* L. (calendula) and *Echinacea purpurea* L. (echinacea), both members of the Asteraceae family, are cultivated principally in Europe, India, and North America [6–9]. *C. officinalis*, whose common name is marigold, is an annual officinal plant, whereas *E. purpurea*, or the common purple coneflower, is a perennial species with medicinal properties. Traditionally used by different cultures for treating mucosal issues, skin conditions, viral infections and digestive disorders, *C. officinalis* and *E. purpurea* are nowadays widely used in the food, food supplement, and cosmetic fields [9, 10].

Fluid extracts of calendula flowers and echinacea roots contain a rich array of bioactive molecules, including phenolic compounds, hydroxycinnamic acids, essential oils, flavonoids, carotenoids, and terpenoids that confer potential health benefits, such as antimicrobial [11, 12], anticancer [13–15] anti‐inflammatory [7, 15], and antioxidant effects [7, 12].

Bacterial fermentation is a well‐established and effective strategy for improving bioavailability of naturally occurring bioactive compounds, lowering substrate pH, and converting raw materials into value‐added functional products [16]. In this context, the fermentation of *C. officinalis* and *E. purpurea* extracts using selected bacterial strains, particularly lactic acid bacteria (LAB), may enhance their antimicrobial and antioxidant potential. Such an approach offers promising opportunities for the development of innovative plant‐based nutraceuticals with improved health benefits.

Despite this potential, studies focusing on the fermentation of calendula and echinacea extracts are scarce, with a single previous study reported on *E. purpurea* extract fermentation [11].

Therefore, the present paper evaluates the capability of some bacterial strains to develop on extracts of *C. officinalis* and *E. purpurea*, aimed at assessing possible synergistic effects and supporting the development of novel fermented plant‐based nutraceuticals with enhanced bioactivity and therapeutic value.

## 2. Materials and Methods

### 2.1. Raw Plant Materials Source and Heat Treatment Definition

Dried *C. officinalis* flowers and *E. purpurea* roots were provided by the company Agripharma Soc. Coop. Agr. (Italy). Raw plant materials were finely ground into a powder using the food processing machine Blendforce glass (Moulinex, Écully, France).

The plant powder was diluted in water at a 1% *w*/*v* ratio and incubated in a water bath under multiple thermal protocols. These consisted of maintaining the samples at 50°C and 60°C (for 2 and 4 h), alongside higher temperature exposures at 65°C (for 30 and 60 min) and 70°C (for 15 and 30 min). The different temperature and duration combinations were selected based on conditions tested by Massaro et al. [17], aimed at identifying a pasteurization treatment capable of reducing the indigenous microbial load in the raw plant material while minimizing heat‐induced degradation of potentially beneficial plant compounds. To evaluate the impact of the heat treatments, aliquots were collected for total microbial count analysis that was performed by plate counting on PCA (Merck, Darmstadt, Germany) and MRS agar (Merck, Darmstadt, Germany) media.

### 2.2. Herbal Extracts Preparation

To prepare the different plant extracts, either 40 g for *C. officinalis* powder or 250 g for *E. purpurea* powder were added to 1 L of preheated water (70°C). Then, samples were incubated at 70°C for 15 min in a water bath, then processed with an Estraggo PRO juice extractor (Siqur Salute, Italy) to remove the solid parts. Aqueous extracts were then centrifuged two times for 10 min at 5300 rpm (Beckman Avanti JXN‐26, Beckman Coulter Inc., Brea, United States). Supernatants were stored frozen at −20°C.

### 2.3. Bacterial Strains and Growth Conditions

Based on the previous screening by Massaro et al. [17], probiotic bacterial strains with robust fermentative capabilities on herbal matrices were selected, namely *Lactiplantibacillus plantarum* 299 V (LACTIfast Rebalance, Marco Viti, Vicenza, Italy), *Pediococcus acidilactici* IRZ12B [18], and *Lacticaseibacillus rhamnosus* GG [19]. Growth capabilities of *Bacillus subtilis* natto (TopCultures, Zoersel, Belgium) were also investigated. LAB strains, stored at −80°C in MRS broth (Merck, Darmstadt, Germany) and *Bacillus* strain, stored in Brain Heart Infusion (BHI) broth (Merck, Darmstadt, Germany), containing 20% (*v*/*v*) glycerol, were activated by incubation at 37°C for 24 h.

### 2.4. Fermentation of Herbal Extracts

Herbal extracts were separately inoculated with approx. A total of 105 CFU/mL of each bacterial strain and incubated at 37°C for 24 h. Total microbial count of the extracts prior to inoculation was assessed by plate counting on PCA at 30°C for 48 h. A noninoculated sample was included as a control to assess possible spontaneous fermentation (SF). Samples were then centrifuged two times for 10 min at a speed of 5300 rpm using a Beckman Avanti JXN‐26 centrifuge. Culture supernatants were transferred to −20°C.

Microbial proliferation was evaluated by comparing aliquots of the inoculated extracts on MRS agar for LAB strains and BHI agar for *Bacillus* at t0 and at t24, that is, after 24 h, upon plate incubation for 48 h at 37°C.

A laboratory bench pH meter was employed to determine the pH of each extract at both the prefermentation and postfermentation stages. (HI5521, Hanna Instruments Inc., Woonsocket, United States).

### 2.5. Minerals

The concentrations of Ca, Fe, Mg, Mn, P, K, Na, and Zn were quantified in raw materials as well as in both fermented and unfermented samples using an ICP‐OES Spectro Arcos system (Spectro Analytical Instruments GmbH, Kleve, Germany), as reported by Massaro et al. [17]. Experiments were repeated three times. Results were given in milligrams per kilogram.

### 2.6. Chemical Analysis

Quantifications of sucrose, D‐fructose, D‐glucose, D‐lactic acid, L‐lactic acid, acetic acid, L‐malic acid, and D‐gluconic acid were carried out using an iCubio iMagic M9 automatic analyzer (R‐Biopharm Italia S.r.l., Melegnano, Italy) using commercial kits from R‐Biopharm (Italy). All measurements were done on both fermented and unfermented samples. Sugar content was also evaluated in the raw material. Data acquisition and processing were done using the iChemMini software (Version 1.0.63.1). Total tannin amount was determined via a colorimetric method from Bate‐Smith [20], with measurements performed on an LT‐4000 microplate reader (LabTech S.r.l., Sorisole, Italy). All analyses were repeated three times and results were reported as grams per liter.

The determination of total phenolic content (TPC) was performed adopting a modified Folin–Ciocalteu assay [21], as indicated by Massaro et al. [17]. Results were reported as mg gallic acid equivalents per mL (mg GAE/mL). All experiments were repeated three times.

### 2.7. High‐Performance Liquid Chromatography (HPLC) Analysis

HPLC analysis was performed with a Shimadzu Nexera XR system (Shimadzu, Kyoto, Japan) equipped with a C18 Kinetex column (4.6 × 150 mm, 5 *μ*m; Phenomenex, Torrance, United States). Detection of phenolic compounds was done at 280 nm using a diode array detector (SPD‐M20A, Waters Corporation, Milford, United States). Individual phenolics were quantified by integrating chromatographic peak areas and comparing them with calibration curves (0.0125–0.2 mg/mL) prepared from commercial standards, including 3,4‐dihydroxycinnamic acid, 4‐hydroxyphenylacetic acid, apigenin, caftaric acid, caffeic acid, caffeine, catechin, chlorogenic acid, chicoric acid, cinnamic acid, ferulic acid, gallic acid, luteolin, phenylacetic acid, protocatechuic acid, quercetin‐3‐glucoside, quercetin aglycone, rosmarinic acid, salicylic acid, sinapic acid, *trans*‐3,4‐dimethoxycinnamic acid, *trans*‐m‐hydroxycinnamic acid, *trans*‐p‐methoxycinnamic acid, vanillic acid, and veratric acid. All solvents and reagents of analytical grade were obtained from Sigma‐Aldrich (St. Louis, United States).

Riboflavin determination was done as indicated by Vendramin et al. [22], using the same chromatographic system and column. Quantification was based on peak area integration and calibration with a standard riboflavin solution.

Data acquisition and processing were done using LabSolutions software (Version 5.93).

All measurements for both phenolic compounds and riboflavin were performed in triplicate.

### 2.8. Antioxidant Activity

The antioxidant capacity of the plant extracts was evaluated by their radical scavenging activity against the stable DPPH^●^ (2,2‐diphenyl‐2‐picrylhydrazyl hydrate), following the procedure described by Miliauskas et al. [23] as modified by Massaro et al. [17].

The percentage of DPPH inhibition was used to determine the antioxidant activity of samples by applying the following formula (*A*_*B*_ is the absorbance of blank, i.e., DPPH solution without extract, and *A*_*S*_ is the absorbance of the tested sample):

%Inhibition=AB−ASAB×100

All tests were repeated three times.

### 2.9. Antimicrobial Activity

*Salmonella enterica* subsp. *enterica* serovar Typhimurium SL1344, *Yersinia enterocolitica* Xen24, and *Escherichia coli* CFA‐I were the pathogenic strains used, obtained from the Norwegian University of Life Sciences (NMBU) (Ås, Norway) culture collection.

Antimicrobial activity assays were conducted following the method of Massaro et al. [17]. Single bacterial colonies were transferred to 5 mL of BHI broth (Oxoid Limited, United Kingdom) and incubated at 37°C for 24 h, then plated on BHI agar to determine viable counts. Cultures were adjusted to approximately 10^5^ CFU/mL in sterile BHI for testing. Antimicrobial assays were conducted in 96‐well microtiter plates, with each well containing 240 *μ*L of bacterial suspension and 60 *μ*L of centrifuged and filtered aqueous plant extract. Blank (with minimum pH values of 5.87 for calendula and 6.04 for echinacea), growth, and sterility controls were included [24].

Plates were stored at 37°C for 12 h, and OD_600_ measurements were taken every 15 min using a Multiskan SkyHigh spectrophotometer (Thermo Fisher Scientific, United States). Antimicrobial activity was given by sample absorbance after subtraction of blank absorbance. All testes were repeated three times.

### 2.10. Statistical Analysis

Data were statistically evaluated using one‐way analysis of variance (ANOVA) followed by Tukey′s post hoc test, performed with XLSTAT software, Version 2016.02 (Addinsoft, Paris, France). Differences were considered statistically significant at *p* < 0.05.

In the antimicrobial assays, growth curves were generated by calculating the averages of absorbances at 600 nm (OD_600_) of three separated experiments. The change in optical density relative to the initial value (*Δ*OD_600_) was calculated to facilitate curve comparison. For each replicate, the area under the growth curve (AUC) was estimated using the trapezoidal rule [25].

## 3. Results and Discussion

### 3.1. Heat Treatment Optimization

Table 1 summarizes the treatment efficacy, expressed as the residual bacterial load, for calendula and echinacea plant materials. In the calendula raw material, starting from an initial microbial population of 6.719 ± 0.191 log CFU/mL, all approaches lowered significantly the microbial load by more than 2 logs. In the echinacea raw material, starting from an initial load of 4.474 ± 0.155 log CFU/mL, the trials at 50°C and 60°C were not effective in reducing the indigenous microbial population. In particular, incubation for 4 h at 50°C provoked an increase close to 10^7^ CFU/mL. In both cases, the treatment at 70°C for 15 min was chosen for its optimal efficacy, as it achieved substantial microbial reduction in a relatively short treatment time, with no significant differences compared with other effective treatments.

**Table 1:** Impact of different heat treatment parameters on the residual microbial counts (log CFU/mL) in calendula and echinacea extracts.

Temperature	Time	Calendula (log CFU/mL)	Echinacea (log CFU/mL)
—	—	6.719 ± 0.191^c*^	4.474 ± 0.155^d*^
50°C	2 h	3.874 ± 0.491^ab^	4.281 ± 0.171^cd^
50°C	4 h	3.874 ± 0.588^ab^	6.804 ± 0.637^e^
60°C	2 h	3.921 ± 0.391^ab^	4.377 ± 0.112^d^
60°C	4 h	3.006 ± 0.864^a^	4.006 ± 0.123^bcd^
65°C	1 h	4.546 ± 0.269^b^	4.025 ± 0.545^bcd^
65°C	30 min	4.456 ± 0.112^b^	3.387 ± 0.144^abc^
70°C	30 min	3.886 ± 0.014^ab^	2.866 ± 0.232^a^
70°C	15 min	3.583 ± 0.443^ab^	3.259 ± 0.241^ab^

*Note:* Values are presented as the mean ± standard deviation (*n* = 3). Different superscript letters within the same row/column indicate statistically significant differences (*p* < 0.05). The asterisk (^*^) denotes the nonheat‐treated control sample.

Pasteurization was utilized rather than sterilization to decrease the natural microbial population within the raw material, thereby limiting the thermal degradation of beneficial plant constituents.

The HTST (high‐temperature short‐time) combination of 70°C for 15 min confirmed its effectiveness in inactivating most indigenous microbial cells while potentially minimizing damage to heat‐sensitive molecules.

This approach is in accordance with that of Bovo et al. [26], which demonstrated that HTST pasteurization achieving more than 2‐log reduction in microbial load effectively mitigates the impact of indigenous microbes on guided fermentation. Consequently, an inoculum of 10^5^ CFU/mL was considered sufficient to supplant the remaining indigenous bacteria in calendula extracts.

### 3.2. Fermentation Trials

The data concerning pH fluctuations and microbial colony formation during the fermentation of calendula and echinacea extracts are presented in Table 2. In calendula extracts, LAB strains grew robustly, showing population increases of approximately 3.5 logs after 24 h, whereas *B. subtilis* natto increased by around 2 logs. Although the spontaneously fermented sample showed no measurable growth on MRS agar (< 10 CFU/mL) throughout the process, the total counts on PCA reached 8.61 ± 0.19 log CFU/mL by the conclusion of the fermentation period. LAB strains significantly reduced the pH of the calendula extract (initial value of the unfermented extract of 5.49 ± 0.085), with decreases ranging from 1.8 units (*L. plantarum* 299 V) to 1.2 units (*L. rhamnosus* GG), whereas *B. subtilis* natto and the spontaneously fermented sample showed negligible pH change.

**Table 2:** Evolution of microbial populations (log CFU/mL) and final pH values in calendula and echinacea extracts. Data are shown at inoculation (*t*0) and following 24 h of fermentation (*t*24) for the four selected strains and a spontaneously fermented control. Initial pH was 5.49 ± 0.085 (calendula) and 5.27 ± 0.045 (echinacea).

	Strain	Log CFU/mL (*t*0)	Log CFU/mL (*t*24)	pH (*t*24)
Calendula	*L. plantarum* 299 V	5.449 ± 0.136	9.032 ± 0.044^c^	3.68 ± 0.025^a^
	*P. acidilactici* IRZ12B	5.270 ± 0.036	8.821 ± 0.027^bc^	4.03 ± 0.040^ab^
	*L. rhamnosus* GG	5.163 ± 0.086	8.310 ± 0.085^b^	4.23 ± 0.011^b^
	*B. subtilis* natto	5.510 ± 0.056	7.566 ± 0.513^a^	5.64 ± 0.295^c^
	SF	< 1	< 1	5.61 ± 0.007

Echinacea	*L. plantarum* 299 V	5.159 ± 0.275	9.288 ± 0.070^c^	4.66 ± 0.005^a^
	*P. acidilactici* IRZ12B	5.091 ± 0.123	9.420 ± 0.021^c^	6.34 ± 0.007^d^
	*L. rhamnosus* GG	5.003 ± 0.187	8.528 ± 0.169^b^	4.64 ± 0.020^a^
	*B. subtilis* natto	5.382 ± 0.332	6.159 ± 0.151^a^	5.47 ± 0.006^c^
	SF	< 1	< 1	5.74 ± 0.007

*Note:* Results are presented as mean ± standard deviation (*n* = 3). Within each column, different lowercase letters denote statistically significant differences (*p* < 0.05).

Abbreviation: SF, spontaneously fermented.

Significant proliferation was observed in LAB strains within the echinacea extracts; notably, *L. plantarum* 299 V and *P. acidilactici* IRZ12B expanded by approximately 4 logs, whereas *L. rhamnosus* GG increased by 3.5 logs. In contrast, *B. subtilis* natto exhibited growth of under 1 log. Although the SF remained undetectable on MRS plates (< 10 CFU/mL), it reached a concentration of 7.85 ± 0.15 log CFU/mL on PCA. Starting from an initial pH of 5.27 ± 0.045, the LAB strains induced a mild acidification, specifically, *L. plantarum* 299 V and *L. rhamnosus* GG lowered the pH by roughly 0.6 units. Conversely, *P. acidilactici* IRZ12B led to a marginal pH increase, and *B. subtilis* natto showed no pH reduction, mirroring the behavior of the control and spontaneously fermented samples. The high growth of LAB in both plant extracts highlights their adaptability to plant‐based substrates.

In fact, these bacterial species have good capability to develop in many plant‐based matrices, successfully tolerating inhibitory compounds commonly found in such substrates, including tannins, lipids, terpenoids, and derivatives of organic acids [10, 27–32]. These observations are consistent with previous studies documenting LAB proliferation in various fruit and vegetable juices [28, 33–35]. In contrast to earlier work by Rizzello et al. [11], that reported poor growth (< 1 log increase) of *L. plantarum* strains in echinacea extracts, our results showed substantial proliferation, with *L. plantarum* 299 V increasing by 4.13 log CFU/mL, likely facilitated by the presence of fermentable carbohydrates.

Although *B. subtilis* natto typically thrives on leguminous substrates [36], it demonstrated robust growth in calendula and other plant extracts [37–39]. However, its proliferation in echinacea was minimal, suggesting that this substrate may lack specific nutrients or present unfavorable conditions.

The pronounced pH reduction during LAB fermentation of calendula aligns with previous studies on other plant matrices [28, 31, 33, 40]. Acidification, driven by organic acid accumulation, is important for inhibiting spoilage and pathogenic microorganisms, thus enhancing both stability and safety of the fermented product [41]. In contrast, *B. subtilis* natto exhibited limited acidification due to its alkaline fermentation profile [42, 43].

In echinacea extracts, LAB strains induced only minor acidification, despite their known acidifying capacity [31, 33, 41]. Moreover, the low growth and negligible pH decrease observed for *B. subtilis* natto underscore the importance of substrate acidification for successful fermentation and microbial control. By acidifying the substrate, inoculated bacteria can effectively inhibit the growth of indigenous microbiota or pathogenic strains, thereby conserving and monopolizing the nutritional components of the substrate exclusively for their proliferation [43]. Limited acidification may reflect a buffering effect from the phytochemical composition of *echinacea*, potentially neutralizing acidic byproducts. Although no direct evidence is available in the literature, it can be hypothesized that specific compounds might interact with acidic byproducts produced during fermentation, neutralizing their effects and stabilizing pH levels. Nevertheless, the precise mechanisms underlying this buffering effect in echinacea remain unclear. Consequently, it is imperative to conduct additional research into the biochemical synergies between echinacea and the metabolic pathways of LAB. Particular attention should be directed toward the *P. acidilactici* IRZ12B strain, given the atypical rise in pH levels observed following the fermentation process.

### 3.3. Mineral Content

As detailed in Table 3, analysis of some minerals was performed on the calendula and echinacea fermented and nonfermented extracts by quantifying the contents of Ca, Fe, Mg, Mn, P, K, Na, and Zn.

**Table 3:** Composition of fermented calendula and echinacea extracts and nonfermented controls in terms of minerals, given in milligrams per kilogram of extract (fresh weight).

	Strain	Ca	Fe	Na	K	Zn	P	Mg	Mn
Calendula	NF	113.235 ± 1.212^a^	0.399 ± 0.007^c^	80.004 ± 0.859^a^	887.464 ± 8.341^a^	1.230 ± 0.021^c^	156.009 ± 1.650^c^	69.899 ± 0.841^a^	0.324 ± 0.003^e^
	*L. plantarum* 299 V	121.718 ± 0.945^b^	0.974 ± 0.014^e^	145.492 ± 0.920^e^	869.139 ± 6.361^a^	1.217 ± 0.017^c^	142.187 ± 0.472^a^	70.957 ± 0.465^a^	0.020 ± 0.000^b^
	*P. acidilactici* IRZ12B	141.850 ± 1.720^c^	0.310 ± 0.008^b^	115.491 ± 0.891^c^	932.174 ± 8.691^b^	0.972 ± 0.017^a^	144.329 ± 1.262^ab^	75.607 ± 0.378^b^	0.010 ± 0.000^a^
	*L. rhamnosus* GG	153.030 ± 0.962^d^	0.702 ± 0.006^d^	108.976 ± 0.315^b^	973.213 ± 8.794^c^	1.030 ± 0.012^b^	153.052 ± 1.724^c^	80.292 ± 0.048^c^	0.027 ± 0.000^c^
	*B. subtilis* natto	149.892 ± 0.963^d^	0.156 ± 0.001^a^	119.381 ± 1.381^d^	966.151 ± 8.798^c^	1.407 ± 0.017^d^	146.034 ± 1.415^b^	79.562 ± 1.049^c^	0.212 ± 0.000^d^

Echinacea	NF	384.804 ± 0.576^c^	2.430 ± 0.030^b^	90.297 ± 0.263^a^	2111.736 ± 11.697^cd^	2.823 ± 0.026^cd^	789.232 ± 0.997^d^	392.796 ± 1.507^c^	0.918 ± 0.008^d^
	*L. plantarum* 299 V	357.438 ± 1.461^a^	6.071 ± 0.038^d^	169.030 ± 0.488^e^	2046.840 ± 9.902^b^	2.901 ± 0.029^d^	803.839 ± 0.552^e^	371.149 ± 0.956^b^	0.017 ± 0.000^a^
	*P. acidilactici* IRZ12B	355.695 ± 2.041^a^	2.212 ± 0.039^a^	155.031 ± 0.879^c^	2115.208 ± 5.662^d^	2.330 ± 0.029^a^	664.966 ± 4.903^b^	351.838 ± 2.775^a^	0.047 ± 0.002^b^
	*L. rhamnosus* GG	399.322 ± 4.979^d^	2.719 ± 0.018^c^	128.386 ± 1.056^b^	1915.921 ± 5.540^a^	2.700 ± 0.035^b^	638.057 ± 4.533^a^	371.623 ± 3.997^b^	0.578 ± 0.012^c^
	*B. subtilis* natto	372.807 ± 2.999^b^	2.641 ± 0.035^c^	159.788 ± 1.085^d^	2088.030 ± 11.527^c^	2.736 ± 0.039^bc^	774.417 ± 3.717^c^	391.791 ± 2.597^c^	0.926 ± 0.011^d^

*Note:* All data are expressed as mean ± standard deviation of triplicates. Different letters in each column indicate statistically significant differences (*p* < 0.05) within the same herbal extract.

Abbreviation: NF, nonfermented.

In terms of the mineral profile of calendula, all fermented extracts displayed a notable rise in Ca concentrations, with *L. rhamnosus* GG achieving an increase of 35%. Fe content followed a more selective pattern, rising significantly only under the influence of *L. plantarum* 299 V and *L. rhamnosus* GG. In contrast, both *P. acidilactici* IRZ12B and *B. subtilis* natto led to a substantial reduction in Fe compared with the control. Na levels, however, were universally boosted by the fermentation process, showing gains ranging from 36% to 80% depending on the strain. K content significantly increased in all the fermented samples, with the sole exception of the fermentation by *L. plantarum* 299 V, where it remained unchanged.

With the exception of *L. plantarum* 299 V, which showed no variation, Zn concentrations were significantly altered across the fermented samples. Specifically, levels declined in extracts treated with *P. acidilactici* IRZ12B and *L. rhamnosus* GG, whereas an upward trend was noted with *B. subtilis* natto. Regarding P, a notable reduction was evident in all fermented groups, specifically those involving *L. plantarum* 299 V, *P. acidilactici* IRZ12B, and *B. subtilis* natto, relative to the control. Similarly, Mg content saw a modest yet statistically significant rise in most extracts, though it remained stable in the *L. plantarum* 299‐V trial.

Fermentation significantly reduced Mn content, particularly in the cases of LAB strains, which were reduced by 91% (*L. rhamnosus* GG) to 96% (*P. acidilactici* IRZ12B).

The mineral composition of echinacea was notably altered during the fermentation process. Although *L. rhamnosus* GG led to an increase in Ca levels, the other three strains resulted in a reduction compared with the initial concentration. Iron dynamics were particularly striking: Although *P. acidilactici* IRZ12B was the only strain to cause a decline, the others spurred an increase. Notably, *L. plantarum* 299 V demonstrated a superior capacity for mineral modulation, tripling the original Fe content, whereas the changes induced by *B. subtilis* natto and *L. rhamnosus* GG were relatively minor.

In all fermented samples, Na content significantly increased from 42% (*L. rhamnosus* GG) to 87% (*L. plantarum* 299 V).

As for K, *L. plantarum* 299 V and *L. rhamnosus* GG were able to significantly decrease it, whereas the extracts fermented by *P. acidilactici* IRZ12B and *B. subtilis natto* did not report statistical differences with respect to the nonfermented sample. However, the extent of the changes was not relevant, even when statistically significant.

Regarding Zn, *P. acidilactici* IRZ12B and *L. rhamnosus* GG decreased it significantly, whereas in the extracts fermented by *L. plantarum* 299 V and *B. subtilis natto*, it remained unchanged.

All changes in P levels following fermentation were statistically relevant, particularly in the case of *L. plantarum* 299 V; an increase was observed, whereas in the case of other strains a decrease was noted. Although significant, the extent of the observed changes was relatively limited.

In all the extracts fermented by LAB strains, Mg content decreased significantly with respect to the nonfermented sample, with the sole exception of the fermentation by *B. subtilis natto* strain, where it remained unchanged. Despite reaching statistical significance, the changes observed were not particularly pronounced.

The fermentation by LAB strains reduced significantly Mn content, particularly in the case of *L. plantarum* 299‐V sample, where 98% was depleted. In contrast, Mn content did not change significantly after fermentation by *B. subtilis natto*.

Minerals are essential for cellular function, enzymatic activity, and structural integrity, making their quantification important for evaluating the nutritional quality of calendula [44–47] and echinacea [48–51] extracts.

Previous studies on calendula plant and extracts reported mineral contents that were both similar and distinct from those observed in the nonfermented sample [45–47]. Moreover, considering our extraction process, mineral contents in the nonfermented echinacea samples can be reputed in line with data presented by Popescu et al. [49] and Çelik and Kan [48] on the raw plant.

The changes in mineral contents after fermentation were similar to those reported for other botanicals [52–55].

The recorded variations in Na, Fe, and P levels in the calendula extract fermented by *B. subtilis natto* were similar to those reported in soybeans fermentation for natto food production [36]. Overall, the *B. subtilis natto* modifications in mineral content were comparable with those observed with the LAB strains.

Higher levels of Na after fermentation accelerate the fermentation process by facilitating the transport of nutrients and active compounds into bacterial cells and improving microbial metabolism and growth efficiency [56].

The observed decrease in Mg content in echinacea fermented extracts might be explained by its importance as a cofactor in various enzymatic reactions, supporting substrate utilization and optimizing bacterial metabolic pathways during fermentation [56].

The impact of fermentation on mineral profiles is characterized by a dual nature: Microbial activity may deplete total mineral concentrations, yet it frequently boosts bioavailability through the dissociation of molecular complexes [54, 57]. This transformation is especially vital for essential elements such as Fe, Zn, and Ca, as the breakdown of phytates during fermentation significantly enhances their absorbability [54]. Furthermore, the metabolic acidification inherent in the process has been shown to optimize the uptake of specific minerals, Fe in particular [54]. However, these outcomes are highly contingent upon the substrate characteristics and preparation. For instance, mechanical treatments like grinding expand the available surface area, thereby promoting microbial engagement and enzymatic efficiency, which in turn improves mineral liberation [57].

### 3.4. Chemical Analysis

The levels of D‐glucose, D‐fructose, sucrose, L/D‐lactic acid, acetic acid, L‐malic acid, gluconic acid, and total tannin for both substrates are detailed in Table 4. In the case of calendula, fermentation led to a drastic depletion of glucose and fructose, with reductions exceeding 88% across all trials. Sucrose utilization varied by strain: Although *L. plantarum* 299 V and B. subtilis natto nearly exhausted the supply, *P. acidilactici* IRZ12B caused only a minor significant decline, and *L. rhamnosus* GG showed no sucrose metabolism. Similarly, echinacea extracts processed with LAB strains saw glucose and fructose levels fall by more than 82% and 96%, respectively. Although *B. subtilis* natto also significantly lowered these sugar concentrations, the extent of the reduction was less pronounced than that observed in the LAB groups.

**Table 4:** Sugars, organic acids, and total tannin content of fermented extracts and nonfermented controls, reported as grams per liter.

	Strain	D‐glucose	D‐fructose	Sucrose	L‐lactic acid	D‐lactic acid	Acetic acid	L‐malic acid	Gluconic acid	Total tannin
Calendula	NF	0.925 ± 0.008^c^	1.771 ± 0.083^b^	1.657 ± 0.009^c^	ND	0.160 ± 0.040^b^	0.219 ± 0.007^a^	1.190 ± 0.003^d^	0.633 ± 0.006^d^	0.170 ± 0.013^c^
	*L. plantarum* 299 V	0.111 ± 0.028^b^	0.082 ± 0.004^a^	0.127 ± 0.144^a^	2.640 ± 0.046^c^	2.518 ± 0.029^d^	0.333 ± 0.027^c^	0.531 ± 0.002^c^	0.523 ± 0.006^c^	0.083 ± 0.014^a^
	*P. acidilactici* IRZ12B	0.099 ± 0.004^b^	0.154 ± 0.007^a^	1.423 ± 0.019^b^	2.063 ± 0.008^b^	1.014 ± 0.016^c^	0.233 ± 0.017^a^	0.511 ± 0.002^b^	0.523 ± 0.006^c^	0.116 ± 0.006^b^
	*L. rhamnosus* GG	0.094 ± 0.007^b^	0.080 ± 0.008^a^	1.640 ± 0.014^c^	2.983 ± 0.007^d^	0.090 ± 0.014^a^	0.373 ± 0.005^d^	0.493 ± 0.006^a^	0.357 ± 0.015^a^	0.121 ± 0.005^b^
	*B. subtilis* natto	0.040 ± 0.016^a^	0.128 ± 0.002^a^	0.117 ± 0.031^a^	0.274 ± 0.008^a^	0.099 ± 0.012^ab^	0.278 ± 0.003^b^	0.534 ± 0.012^c^	0.457 ± 0.021^b^	0.098 ± 0.006^ab^
	NF	0.570 ± 0.011^d^	3.317 ± 0.013^c^	10.223 ± 0.383^d^	0.009 ± 0.008^a^	0.251 ± 0.017^b^	0.282 ± 0.009^a^	2.286 ± 0.012^d^	0.370 ± 0.035^ab^	2.809 ± 0.233^c^

Echinacea	*L. plantarum* 299 V	0.141 ± 0.013^b^	0.097 ± 0.003^a^	8.123 ± 0.039^b^	3.798 ± 0.012^c^	4.161 ± 0.043^d^	1.559 ± 0.030^e^	0.516 ± 0.007^a^	0.310 ± 0.010^a^	1.864 ± 0.074^ab^
	*P. acidilactici* IRZ12B	0.083 ± 0.025^a^	0.085 ± 0.006^a^	8.879 ± 0.069^c^	3.869 ± 0.032^d^	2.749 ± 0.022^c^	0.812 ± 0.008^d^	0.530 ± 0.008^ab^	0.373 ± 0.021^b^	1.811 ± 0.201^a^
	*L. rhamnosus* GG	0.135 ± 0.001^b^	0.113 ± 0.006^a^	8.468 ± 0.149^bc^	5.308 ± 0.015^e^	0.266 ± 0.011^b^	0.640 ± 0.025^c^	0.550 ± 0.016^b^	0.313 ± 0.006^ab^	2.401 ± 0.256^bc^
	*B. subtilis* natto	0.351 ± 0.007^c^	2.541 ± 0.127^b^	6.342 ± 0.053^a^	0.075 ± 0.018^b^	0.086 ± 0.030^a^	0.352 ± 0.020^b^	1.948 ± 0.011^c^	0.450 ± 0.030^c^	1.987 ± 0.199^ab^

*Note:* Data are presented as mean ± standard deviation (*n* = 3). Different lowercase letters within the same column and for the same botanical extract indicate statistically significant differences (*p* < 0.05).

Abbreviations: ND, not detected; NF, nonfermented.

Sucrose initial content in the nonfermented extract was largely higher with respect to glucose and fructose levels. All fermented samples significantly decreased sucrose content, with a maximum reduction of 37% (*B. subtilis natto*).

Sugar content of the calendula and echinacea nonfermented extracts was consistent with earlier findings [51, 58–60]. During fermentation, these sugars content was significantly reduced, similar to what previously observed in various plant extracts and juices fermented with strains of the same bacterial species [33, 35, 40, 42, 61]. Indeed, it is known that LAB utilize glucose and fructose as fermentation substrates. *L. plantarum* 299 V and *B. subtilis natto* also metabolized sucrose, with the second one demonstrating the highest consumption level in the echinacea matrix, compared with the LAB strains.

Regarding organic acid profiles in calendula, L‐lactic acid, initially absent in the raw extract, reached high concentrations following LAB fermentation, whereas *B. subtilis* natto produced only moderate levels. Interestingly, the baseline level of D‐lactic acid in the nonfermented sample exceeded that of the L‐isomer. Although *L. rhamnosus* GG and *B. subtilis* natto successfully reduced D‐lactic acid content, *L. plantarum* 299 V and *P. acidilactici* IRZ12B synthesized substantial quantities, with the former producing more than twice the amount of the latter. Acetic acid levels rose across nearly all trials, with *P. acidilactici* IRZ12B being the only strain to show no change. Furthermore, a consistent reduction in L‐malic acid was observed, with depletion rates ranging from 55% to 58% across all fermented extracts. Regarding gluconic acid, its content was significantly reduced following fermentation, by up to 43% (*L. rhamnosus* GG).

In echinacea, L‐lactic acid was entirely absent prior to fermentation but reached significantly elevated levels across all processed samples. The most substantial accumulation was recorded in the *L. rhamnosus* GG trial, whereas *L. plantarum* 299 V and *P. acidilactici* IRZ12B also contributed to its synthesis. In the unfermented state, D‐lactic acid concentrations actually exceeded those of the L‐isomer. Postfermentation, *L. plantarum* 299 V and *P. acidilactici* IRZ12B yielded high D‐lactic levels, with the former producing roughly 1.5 times the amount of the latter. Interestingly, although *L. rhamnosus* GG was the top producer of L‐lactic acid, it generated the smallest quantity of the D‐isomer among the LAB group. Although *B. subtilis* natto led to statistically significant gains in both acid forms, its overall production remained lower than that achieved by the LAB.

Acetic acid levels rose significantly across all fermentation trials, though the degree of accumulation was highly strain‐specific. The most substantial gains were attributed to *L. plantarum* 299 V and *P. acidilactici* IRZ12B, which achieved more than fivefold and nearly threefold increases, respectively, relative to unfermented control. Regarding L‐malic acid, its initial concentration was drastically reduced by the LAB strains, with depletion rates exceeding 76%. In contrast, the reduction observed during *B. subtilis* natto fermentation was considerably more modest, reaching approximately 15%.

Gluconic acid remained at a stable level following the fermentation by LAB strains, whereas after the fermentation of *B. subtilis natto*, a significant increase was observed.

Lactic acid serves as a fundamental metabolite in fermentation, playing a critical role in the acidification of the substrate [40, 61]. In this study, both L‐ and D‐isomers were monitored to distinguish between general lactic fermentation and malolactic pathways [62–64], the latter of which was further evaluated by tracking L‐malic acid concentrations [62, 65]. Furthermore, gluconic and acetic acids were quantified as secondary markers of microbial metabolism [16, 66]. Consistent with previous literature on echinacea [67] and calendula [58, 59, 68], the initial absence of acetic and lactic acids in the raw extracts confirms that their subsequent detection is exclusively a result of bacterial synthesis.

In fact, fermentation by LAB significantly increased lactic acid concentration in calendula and echinacea extracts, reflecting an efficient carbohydrate metabolism, in line with prior research on various plant‐based substrates fermented by *L. plantarum* and *L. rhamnosus* [11, 33, 41, 61–63].

The acetic acid yields observed in this study underscore the metabolic flexibility of *L. plantarum* and *L. rhamnosus*, both of which are facultative heterofermentative organisms. Conversely, *P. acidilactici* IRZ12B and *B. subtilis* natto displayed a limited capacity for acetic acid synthesis, providing only negligible contributions to the final concentrations.

The low production of organic acids in the extract fermented by *B. subtilis natto* was coherent with the lack of acidification and reflects the metabolism of this strain that does not lower the pH of the matrix [42, 43].

The depletion of L‐malic acid in both echinacea and calendula extracts aligns with existing research on various botanical substrates [30, 33, 34, 62, 69]. This trend suggests the occurrence of malolactic fermentation, a pathway in which malic acid undergoes enzymatic transformation into L‐lactic acid. The activation and scale of this metabolic route are highly contingent upon the specific microbial strain and the prevailing fermentation parameters [70]. In particular, *L. plantarum* and *L. rhamnosus* are recognized for their ability to metabolize malic acid as an auxiliary carbon source [33].

Unchanged or decreased gluconic acid levels in echinacea and calendula fermented samples, respectively, aligned with previous studies, which describe how LAB‐driven fermentation can lead to stabilization or degradation of gluconic acid, depending on the metabolic pathways involved [66, 71–73]. In this study, LAB strains metabolized gluconic acid rather than producing it.

In calendula extract fermented by *B. subtilis natto*, a reduction in malic acid, gluconic acid, and tannins was noted, comparable with that obtained by LAB strains activity.

In the case of echinacea extracts, the scarce contribution of *B. subtilis natto* to organic acids content is evidenced by the pH level of the sample. Limited lactic and acetic acid production aligned with the metabolic traits of *B. subtilis natto*, which do not metabolically rely on acidification [42, 43]. Instead, *B. subtilis natto* normally favors metabolic pathways leading to the production of neutral or alkaline compounds, such as ammonia derived from protein degradation, and may even result in pH increase during fermentation [42].

Tannin levels in the fermented extracts were analyzed to evaluate whether the microbial activity can have an effect on them [60, 74].

A substantial depletion of total tannin was observed across all fermented calendula extracts; most notably, the *L. plantarum* 299‐V strain achieved a 51% decrease in tannin concentration.

In echinacea extracts, total tannin content was significantly reduced by approximately 29%–35% in all fermented samples, except for the one fermented with *L. rhamnosus* GG, which showed a not statistically significant 14% reduction.

The observed reduction in tannin levels in fermented extracts is likely linked to bacterial enzymatic activities, particularly to the presence of tannase. In fact, tannase activity, documented in the species used in this study [54, 75–77], leads to the breakdown of complex tannins during fermentation and yields smaller molecules with increased bioavailability and enhanced antioxidant properties [75].

Moreover, tannins can also be considered as potential antinutritional factors due to their ability to bind to metal ions, thus limiting the extent to which key minerals can be absorbed and utilized by the organism, such as for the case of Fe, thus inhibiting the activity of metal‐dependent enzymes [75, 78]. In this manner, the reduction in tannin content during fermentation may not only mitigate such antinutritional effects but also enhance other bioactive properties resulting from tannin degradation [75].

**Figure 1:**
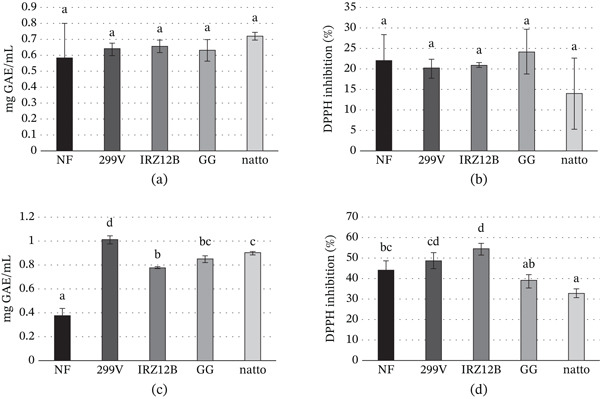
Comparison of phytochemical and functional properties of fermented and nonfermented (NF) herbal extracts. (A) Total phenolic content (mg GAE/mL) and (B) antioxidant activity (% DPPH inhibition) of *Calendula officinalis* extracts. (C) Total phenolic content (mg GAE/mL) and (D) antioxidant activity (% DPPH inhibition) of *Echinacea purpurea* extracts. Different lowercase letters indicate statistically significant differences (*p* < 0.05) within the same assay. NF: nonfermented control.

Variations in the TPC of calendula extracts, comparing fermented samples with their nonfermented counterparts, are detailed in Figure 1A. None of the fermented samples reported significant differences with respect to the nonfermented extract.

TPC represents a fundamental information for assessing the phenols profile of plant extracts [28, 79]. TPC of nonfermented calendula extract was consistent with Santos Ferreira et al. [80] findings, whereas unchanged values after fermentation were in line with Li et al. [81] on apple juice that was fermented by *L. plantarum*.

The TPC for echinacea extracts is illustrated in Figure 1C. Each fermentation trial prompted a substantial rise in GAE/mL concentrations, effectively doubling the levels found in the unfermented control. Of particular interest was the *L. plantarum* 299‐V trial, where the TPC increased nearly threefold relative to the baseline. This general upward trend aligns with prior research involving various botanical matrices and juices fermented with *Lactobacillus*, *Pediococcus*, and *Bacillus* species [28, 31, 40, 82–86]. The strain‐specific fluctuations in TPC likely stem from diverse metabolic efficiencies and the varying production of hydrolytic enzymes. These enzymes are responsible for the biotransformation of complex phytochemicals, liberating insoluble or conjugated phenols from the plant matrix and converting them into simpler, more bioavailable constituents [81, 83, 84].

### 3.5. Polyphenols and Vitamin Analysis

HPLC revealed a diverse profile of peaks, corresponding to the intricate phytochemical fingerprints inherent in both calendula and echinacea extracts.

In calendula samples, only chlorogenic, caftaric, protocatechuic acid, and quercetin glucoside were identified (Table 5).

**Table 5:** Results from HPLC analysis of calendula and echinacea extracts of chlorogenic acid, caftaric acid, protocatechuic acid, quercetin glucoside, caffeine, and riboflavin content, reported as micrograms per milliliter.

	Strain	Chlorogenic acid	Caftaric acid	Protocatechuic acid	Quercetin glucoside	Caffeine	Riboflavin
	NF	110.781 ± 0.206^c^	17.312 ± 0.235^b^	18.020 ± 0.060^c^	222.533 ± 0.107^c^	ND	0.339 ± 0.002^c^
	*L. plantarum* 299 V	71.499 ± 0.015^a^	10.940 ± 0.018^a^	12.682 ± 0.028^a^	192.237 ± 1.519^a^	ND	0.024 ± 0.003^a^
Calendula	*P. acidilactici* IRZ12B	100.264 ± 0.035^b^	16.982 ± 0.065^b^	15.372 ± 0.012^b^	215.926 ± 0.183^b^	ND	0.316 ± 0.021^c^
	*L. rhamnosus* GG	115.347 ± 0.592^e^	16.890 ± 0.267^b^	17.506 ± 0.884^c^	223.464 ± 0.302^c^	ND	0.231 ± 0.001^b^
	*B. subtilis* natto	112.934 ± 0.084^d^	18.446 ± 0.054^c^	17.920 ± 0.017^c^	227.898 ± 0.193^d^	ND	0.369 ± 0.001^d^
	NF	ND	ND	ND	ND	17.031 ± 0.264^d^	0.678 ± 0.004^c^
	*L. plantarum* 299 V	ND	ND	ND	ND	14.335 ± 0.159^b^	0.294 ± 0.001^a^

Echinacea	*P. acidilactici* IRZ12B	ND	ND	ND	ND	4.278 ± 0.564^a^	0.768 ± 0.049^d^
	*L. rhamnosus* GG	ND	ND	ND	ND	17.472 ± 0.299^d^	0.413 ± 0.001^b^
	*B. subtilis* natto	ND	ND	ND	ND	15.407 ± 0.037^c^	0.630 ± 0.002^c^

*Note:* Results are expressed as mean ± SD (*n* = 3). Different letters in the same column indicate significant statistical differences (*p* < 0.05) between treatments of the same plant species.

Abbreviations: ND, not detected; NF, nonfermented.

Fermentation significantly affected chlorogenic acid content in all samples, particularly in *L. rhamnosus* GG and *B. subtilis natto* by increasing it and in *L. plantarum* 299 V and *P. acidilactici* IRZ12B by decreasing it.

Caftaric acid content was significantly modified only after growth with *L. plantarum* 299 V and *B. subtilis natto;* specifically, in the former, a marked decrease was observed, whereas in the second one, a slight increase was recorded.

As for protocatechuic acid, only *L. plantarum* 299 V and *P. acidilactici* IRZ12B were able to significantly decrease its content (29% and 15%, respectively).

Fermentation by *L. rhamnosus* GG did not affect the quercetin glucoside levels. Conversely, other fermented samples resulted significantly different compared with the nonfermented extracts, especially *L. plantarum* 299 V and *P. acidilactici* IRZ12B that led to a significant quercetin decrease, whereas *B. subtilis natto* induced a slight increase.

Protocatechuic, chlorogenic, caftaric acid, and quercetin glucoside, recognized for their health‐related properties, have also been previously identified in calendula [44, 87, 88].

Chlorogenic acid and quercetin contents of the nonfermented extracts were consistent with Santos Ferreira et al. [80] findings. Results of the same molecules in the extract fermented by *B. subtilis natto* aligned with those of Hu et al. [82] on fermented rose extract. This widespread decrease across the measured components in calendula aligns with reported trends in various botanical matrices and fruit‐derived substrates processed with *L. plantarum* and *L. rhamnosus* strains [33, 84, 85].

Levels of phenolic compounds after fermentation are indeed influenced by bacterial strain, enzymatic system, nutrient availability, and intrinsic characteristics of plant material [84]. Variations in phenols content reflect the enzymatic potential of LAB to modify or degrade phenolic structures [84]. For example, the chemical structure of quercetin presents five hydroxyl groups, conferring significant antioxidant potential. However, its glycosylated derivative lowers the antioxidant effect with respect to its aglycone form [89]. On the other hand, bacterial enzymatic activities not only detoxify plant matrix but may also increase bioavailability of phenolics by breaking down complex molecules into simpler, more bioactive forms [90, 91]. This bioconversion during growth enhances the functional properties of plant‐based extracts, potentially improving bioavailability and bioactivity of phenolic compounds [90, 91].

Regarding polyphenols of echinacea samples, only caffeine was identified (Table 5). Fermentation significantly altered the caffeine levels in the initial extract, most notably through the activity of *P. acidilactici* IRZ12B, which achieved a reduction of roughly 75%. In contrast, *L. rhamnosus* GG proved to be the exception, as its growth resulted in no measurable change to the caffeine concentration.

Differently from what is reported in the existing literature, caffeine was found in echinacea extracts. Caffeine identified in the samples is likely a derivative of echinacoside, a prominent bioactive compound present in *E. purpurea* known for its pharmacological benefits, such as neuroprotective and cardiovascular activities [92]. As a major plant alkaloid [93], caffeine acts as a central nervous system stimulant with well‐documented cognitive and antioxidant benefits. However, its widespread use is tempered by the risk of side effects, most notably anxiety, tachycardia, and insomnia, which occur when consumption thresholds are exceeded. These adverse outcomes necessitate caution, particularly for patient groups who may be predisposed to caffeine sensitivity [93].

The reduction of caffeine levels through fermentation represents a potential strategy for managing the ambivalent nature of this compound. This bioprocess could lower caffeine content while preserving other beneficial properties of echinacea extract, broadening its accessibility to individuals sensitive to this molecule or to those who need to avoid it for health reasons.

Riboflavin levels were quantified in both calendula and echinacea samples (Table 5).

In the case of calendula, the riboflavin content increased significantly only after fermentation by *B. subtilis natto*, whereas all the other samples showed a decrease, with *L. plantarum* 299 V reducing it by approximately 93%.

In the case of echinacea, variations in riboflavin levels after fermentation were statistically significant, with the sole exception of *B. subtilis natto*, where there were no significant differences with respect to the nonfermented material. Specifically, fermentation by *L. plantarum* 299 V and *L. rhamnosus* GG induced a decrease, whereas *P. acidilactici* IRZ12B increased its content.

Riboflavin is a water‐soluble vitamin (B2) essential for human health and it is naturally present in many herbs [51, 94]. The observed decrease in riboflavin quantities aligned with previous research on some *L. plantarum* and *L. rhamnosus* strains that are known to be able to consume vitamin B2 [94–96]. Conversely, *P. acidilactici* IRZ12B did not affect riboflavin content in calendula extract, whereas it increased in echinacea extract, indicating its potential ability to either synthesize or preserve this vitamin [95, 97].

On the other hand, unchanged or increased riboflavin levels observed in the extracts fermented by *B. subtilis natto* agreed with earlier reports on soybean fermentation [36], confirming the reported capability of various *Bacillus* species to preserve or produce vitamins [36, 98].

### 3.6. Antioxidant Activity

The DPPH radical scavenging capacity of both fermented and unfermented calendula extracts is detailed in Figure 1B. Statistical analysis revealed that the antioxidant activity remained stable postfermentation, with no significant deviations observed across the various strains. The baseline antioxidant values for the raw extract align with those reported by Mubashar Sabir et al. [87] and Chamansara et al. [99]. Furthermore, the maintenance of these levels throughout the fermentation process parallels the observations of Li et al. [81], who noted similar stability in apple juice processed with *L. plantarum*.

The neutralization of free radicals represents a fundamental pathway by which antioxidants counteract oxidative degradation [28, 100]. The stability observed in antioxidant activity values was coherent with the TPC results, in accordance with previous findings suggesting a correlation between the two parameters [89, 101]. The stability of total phenolics values after fermentation ensures the preservation of radical scavenging activity, as they are key contributors to the antioxidant power. Contrary, the radical scavenging activity of calendula extracts can be linked to the presence of flavonoids such as quercetin, which exhibits a more pronounced antiradical activity compared with phenolic, hydroxybenzoic, and hydroxycinnamic acids [12, 89]. Therefore, antioxidant activity results agree also with the observed reduction in polyphenols, especially quercetin. Flavonoids and phenols of calendula act as reducing agents and metal chelators, modulating oxidative stress and contributing to the overall antioxidant activity of plant extracts [12, 89].

The antioxidant activity results on fermented and nonfermented echinacea extracts are shown in Figure 1D. The changes observed in the fermented samples were variable among strains, where only *P. acidilactici* IRZ12B and *B. subtilis natto* produced significant differences: the first one by increasing and the second one by decreasing the antioxidant activity.

Antioxidant activity results of the nonfermented echinacea extract were consistent with Hu and Kitts [102] findings on fermented rose extract. Results of the extract fermented by *B. subtilis natto* were coherent with Chou et al. [103] findings on natto extract.

The elevated antioxidant capacity recorded in all LAB‐fermented samples is consistent with earlier reports investigating vegetal extracts and fruit juices processed with various *L. plantarum*, *P. acidilactici*, and *L. rhamnosus* strains [28, 35, 41, 83, 85, 104, 105]. This trend likely reflects the established positive correlation between antioxidant potency and total phenolic levels, reinforcing the role of phenolic constituents as major drivers of the radical scavenging strength in botanical extracts [63, 101].

### 3.7. Antimicrobial Activity

In terms of antimicrobial activity, the methodology used in this study assessed whether LAB‐fermented plant extracts could affect pathogens′ growth kinetics by measuring the optical density of the growing bacterial culture.

**Figure 2:**
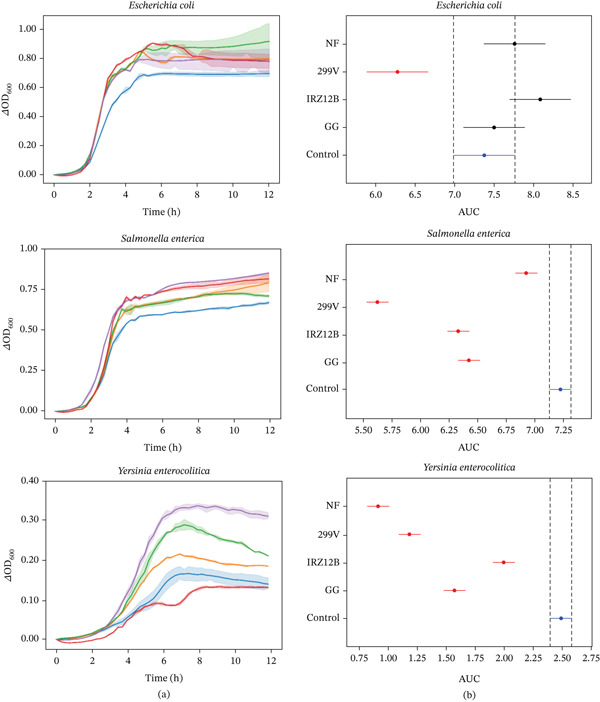
Antimicrobial impact of calendula extracts on indicator bacterial strains. (A) Growth kinetics displayed as change in optical density (*Δ*OD_600_) over time. Curves represent the mean of replicates, with shaded areas indicating the standard deviation (SD) for *L. plantarum* 299 V (▬), *P. acidilactici* IRZ12B (▬), *L. rhamnosus* GG (▬), NF (▬), and control (▬). (B) Statistical comparison of growth inhibition based on area under the curve (AUC) values. The graph displays 95% confidence intervals derived from Tukey′s HSD test. Red bars denote significant differences relative to the control (blue bar), whereas grey bars indicate nonsignificance. Overlapping intervals signify a lack of statistical difference. NF: nonfermented; control: indicator bacterial suspension.

As shown in Figure 2, the impact of calendula extracts on *E. coli* varied by strain; although *P. acidilactici* IRZ12B and *L. rhamnosus* GG showed negligible inhibitory activity, *L. plantarum* 299 V provided a significant reduction in pathogen growth. These results highlight the strain‐specific nature of the antimicrobial enhancement following fermentation.

Conversely, growth of *S. enterica* and *Y. enterocolitica* was significantly reduced in all the tested samples, with a more pronounced inhibitory effect observed against the second one.

Previous studies documented the antimicrobial effects of calendula extracts against various *E. coli*, *S. enterica*, and *Y. enterocolitica* strains, in terms of a significant growth inhibition [106–108]. Our study did not detect inhibitory activity against the tested *E. coli* strain; instead, it aligns with the effectiveness reported in earlier investigations on *S. enterica* and *Y. enterocolitica*. Although the nonfermented extract exhibited greater antimicrobial activity against *Y. enterocolitica* with respect to the fermented samples, in the case of *S. enterica* the fermented ones outperformed the nonfermented counterpart. Calendula contains many compounds endowed with biological activity, including flavonoids, terpenes, and phenolic acids, such as chlorogenic acid, which possess antimicrobial properties [108, 109].

**Figure 3:**
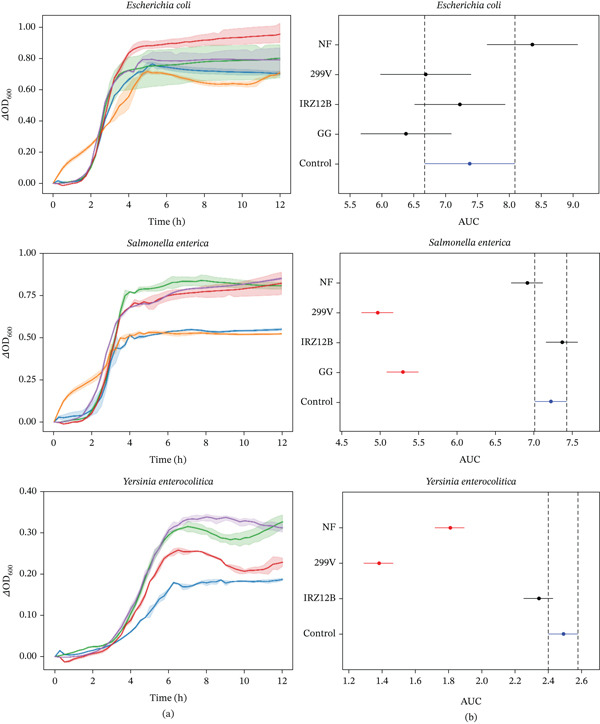
Antimicrobial effects of echinacea extracts on indicator bacterial strains. (A) Growth kinetics represented by change in optical density (*Δ*OD_600_) over time. Solid lines indicate the mean, whereas shaded areas denote the standard deviation (SD) for *L. plantarum* 299 V (▬), *P. acidilactici* IRZ12B (▬), *L. rhamnosus* GG (▬), NF (▬), and control (▬). **(**B**)** Statistical analysis of indicator strain growth based on area under the curve (AUC) values. The plot displays 95% confidence intervals from Tukey′s HSD test. Red bars signify a statistically significant difference relative to the control (blue bar), whereas grey bars indicate no significant variation. Overlap between intervals indicates statistical equivalence. NF: nonfermented; control: indicator bacterial suspension.

The antimicrobial efficacy of the echinacea extracts is detailed in Figure 3. In assays against *E. coli*, neither the fermented nor the unfermented samples provided a statistically significant decrease in bacterial growth. However, response patterns varied for *S. enterica*, which was significantly inhibited only by extracts processed with *L. plantarum* 299 V and *L. rhamnosus* GG; conversely, the raw extract and the *P. acidilactici* IRZ12B fermented samples lacked potency against this pathogen. Regarding *Y. enterocolitica*, growth was successfully suppressed by the nonfermented sample and most notably by the *L. plantarum* 299‐V extract, which exerted the most substantial inhibitory effect. In contrast, *P. acidilactici* IRZ12B had no impact on *Y. enterocolitica* kinetics. Due to a lack of reproducibility, data regarding the influence of *L. rhamnosus* GG on *Y. enterocolitica* were excluded from the analysis.

The antimicrobial potential of *Echinacea* was reported in several papers, demonstrating inhibitory effects against many pathogens, including *E. coli* and *Y. enterocolitica* [7, 13, 29]. For instance, in a similar study of Rizzello et al. [11] echinacea aqueous extract fermented by different strains of *L. plantarum* showed significant inhibitory activity on the growth of both pathogens. Other investigations reported the inhibition of *Salmonella* Enteritidis and *S.* Typhimurium growth by various echinacea extracts [13, 110]. Our findings did not confirm these studies in the case of our *E. coli* strain, whereas for *S. enterica* and *Y. enterocolitica*, the observed antimicrobial activity was consistent with previously published research. Specifically, the extract fermented by *L. plantarum* 299 V demonstrated relevant inhibition on both pathogens, whereas samples fermented by *P. acidilactici* IRZ12B lacked similar efficacy. The nonfermented echinacea extract significantly slowed down the growth of *Y. enterocolitica,* but had no impact on *S. enterica*.

It is known from the literature that *E. purpurea* produces various bioactive compounds, including alkylamides, cichoric, chlorogenic, and caffeic acids, along with their derivatives, that demonstrate antimicrobial properties [7, 29, 111]. Moreover, the enhanced TPC after fermentation not only boosts the antioxidant power but may further increase the antimicrobial potential of echinacea extracts [91, 101].

The enhanced antimicrobial potency of the fermented calendula and echinacea extracts is likely driven by the accumulation of organic acids, primarily lactic and acetic acid, synthesized during the LAB fermentation process. Furthermore, the secretion of specialized antimicrobial metabolites by these bacteria may further contribute to the inhibitory environment [30, 112, 113]. These observations are corroborated by earlier research, which documented superior growth suppression of *E. coli* and *S.* Typhimurium in fruit juices fermented with identical bacterial species [35, 40, 109, 114].

## 4. Conclusions

The present study confirms that fermentation with selected LAB and *B. subtilis* strains is an effective strategy for transforming the chemical landscape of *C. officinalis* and *E. purpurea*. Beyond successfully colonizing the herbal substrates and lowering pH, these microorganisms drove the depletion of tannins and the enrichment of bioavailable minerals. Most notably, the process yielded extracts with markedly enhanced antioxidant and antimicrobial properties alongside higher TPC. These outcomes underscore the transformative power of fermentation to optimize the bioactive characteristics of herbal extracts. Consequently, these results offer a strong foundation for scaling up production and exploring the use of these fermented botanicals in the pharmaceutical and functional food sectors to target chronic health conditions.

## Author Contributions

**Viviana Corich:** conceptualization, resources, writing – review and editing, funding acquisition, project administration. **Davide Porcellato:** conceptualization, writing – review and editing. **Alessio Giacomini:** conceptualization, resources, writing – original draft preparation, writing – review and editing, supervision, funding acquisition, project administration. **Sofia Massaro:** formal analysis, investigation, writing – original draft preparation, writing – review and editing. **Jacopo Sica:** formal analysis, investigation. **Gloria Ghion:** formal analysis, investigation. **Chiara Nadai:** formal analysis, investigation, writing – original draft preparation, writing – review and editing. **Simone Vincenzi:** formal analysis, investigation.

## Funding

This study was supported by the Programma Operativo Nazionale Ricerca e Innovazione 2014–2020. Open access publishing facilitated by Universita degli Studi di Padova, as part of the Wiley ‐ CRUI‐CARE agreement.

## Conflicts of Interest

The authors declare no conflicts of interest.

## Data Availability

The data that support the findings of this study are available from the corresponding author upon reasonable request.
